# Whole genome-based reclassification of several species of the genus *Nonomuraea*

**DOI:** 10.1371/journal.pone.0327003

**Published:** 2025-07-01

**Authors:** Noureddine Bouras, Imen Nouioui, Guendouz Dif, Fawzia Chaabane Chaouch, Sherif S. Ebada

**Affiliations:** 1 Laboratoire de Valorisation et Conservation des Ecosystèmes Arides (LVCEA), Faculté des Sciences de la Nature et de la Vie et Sciences de la Terre, Université de Ghardaia, Ghardaïa, Algeria; 2 Laboratoire de Biologie des Systèmes Microbiens (LBSM), Ecole Normale Supérieure de Kouba, Algiers, Algeria; 3 Department of Bioeconomy and Health Research, Leibniz-Institut DSMZ – German Collection of Microorganisms and Cell Cultures, Braunschweig, Germany; 4 École Normale Supérieure Taleb Abderrahmane de Laghouat, Département des Sciences Naturelles, Laghouat, Algeria; 5 Department of Pharmacognosy, Faculty of Pharmacy, Ain Shams University, Cairo, Egypt; Universidad Nacional Autonoma de Mexico Facultad de Quimica, MEXICO

## Abstract

Earlier classification methods lacked the resolution necessary to delineate species comparing to current standards, particularly since a significant number of bacterial taxa were described before the integration of whole-genome sequencing into microbial taxonomy. With the advent of genome-based taxonomic approaches and integrated genomic metrics, including sequence similarity and phylogenomic frameworks, it has become essential to reevaluate the taxonomic status of older taxa defined before the era of genome-based microbial taxonomy. Applying these modern genomic criteria enables a more accurate assessment of species boundaries and helps to determine whether the old taxa represent distinct species or should be reclassified as synonyms, subspecies, or members of different genera. Such reclassification efforts are crucial for ensuring taxonomic accuracy, improving the reliability of comparative genomics, and maintaining stable and evolutionariy meaningful nomenclature. In this study, we re-evaluated the taxonomy of *Nonomuraea* using genome-based methods, including digital DNA-DNA hybridization (dDDH), average nucleotide identity (FastANI), average amino acid identity (AAI), and percentage of conserved proteins (POCP) analyses, complemented by phylogenomic analysis. Based on comprehensive genomic studies, our results support the elevation of both *Nonomuraea roseoviolacea* subsp. *carminata* and *Nonomuraea roseoviolacea* subsp. *roseoviolacea* to an unified species without subspecies distinction: *Nonomuraea roseoviolacea* (ATCC 27297^T^ = BCRC 13406^T ^= CBS 260.72^T^ = CCM 3491^T ^= CCRC 13406^T^ = CGMCC 4.1072^T^ = CIP 106924^T^ = DSM 43144^T ^= IFO 14098^T^ = IMET 9751^T^ = JCM 3145^T^ = KCTC 9283^T ^= NBRC 14098^T^ = NCIB 11117^T^ = NCIMB 11117^T^ = NRRL B-16127^T^ = VKM Ac-909^T^). Furthermore, we propose the reclassification of *Nonomuraea recticatena*, *Nonomuraea roseola* and *Nonomuraea dietziae* as subspecies within *N. roseola*. Consequently, we propose the establishment of the following two subspecies: *Nonomuraea roseola* subsp. *roseola* subsp. nov. (= ATCC 33579^T^ = DSM 43767^T^ = DSM 44323^T^ = IFO 14685^T^ = IMET 9576^T^ = INA 1671^T^ = JCM 3323 ^T^ = KCTC 9282^T^ = NBRC 14685^T^ = VKM Ac-1180^T^) and *Nonomuraea roseola* subsp. *recticatena* subsp. nov., comb. nov. (= DSM 43937^T ^= IFO 14525^T^ = INA 308^T^ = JCM 6835^T^ = KCTC 9279^T^ = NBRC 14525^T^ = VKM Ac-940^T^) and. In addition, we propose *Nonomuraea harbinensis* as later heterotypic synonyms of *Nonomuraea ferruginea.*

## Introduction

The genus *Nonomuraea* belongs to the family *Streptosporangiaceae* and order *Streptosporangiales*, first proposed by Zhang et al. [1] with *Nonomuraea pusilla* as the type species. Members of this genus are aerobic, Gram-positive, non-acid-fast, non-motile *Actinomycetota* that form extensively branched substrate and aerial mycelia. As of this writing, 68 species and two subspecies of *Nonomuraea* have been validly named (https://lpsn.dsmz.de/genus/nonomuraea, accessed on 5 may 2025). Additionally, several new species have been described in recent years, including *Nonomuraea aridisoli* [2], *Nonomuraea aurantiaca* [3], *Nonomuraea basaltis* [4], *Nonomuraea cypriaca* [5], *Nonomuraea montanisoli* [6], *Nonomuraea rhizosphaerae* [7], *Nonomuraea sediminis* [8], and Nonomuraea *terrae* [9].

*Nonomuraea* is renowned for its diverse metabolic capabilities and potential applications in biotechnology and medicine. Recent studies have identified novel strains within this genus, highlighting their capacity to produce various secondary metabolites with antimicrobial and anticancer properties. These include the glycopeptide antibiotic A40926 [10]; myxochelin A [11]; anthelmintic macrolactams such as fluvirucin B0, Sch 38516/fluvirucin B1, and Sch 39185/fluvirucin B3 [12]; and novel cyclic tetrapeptides such as WSS2220 [13,14]. The strain KC-061^T^, isolated from southern Thailand, has also been reported to produce maduramycin [15] and madurahydroxylactone [16]. Additionally, pradimicin U, a compound with antimicrobial activity, has been identified in *Nonomuraea composti* [17]. *Nonomuraea jiangxiensis* has been recognized as a source of nine new thiopeptides exhibiting strong antimicrobial activity against Gram-positive bacteria, particularly *Staphylococcus aureus* [18]. Moreover, a deep-sea strain of *Nonomuraea* has been found to produce new enediyne antibiotics, sealutomicins, which exhibit potent activity against carbapenem-resistant *Enterobacteriaceae* [19].

*Nonomuraea* species are widely distributed in both terrestrial and aquatic ecosystems [20] and are primarily isolated from soil, but they are increasingly found in varied ecological niches, including coastal and desert environments [2,21,22]. Like other soil bacteria, these species play significant roles in ecological and agricultural contexts due to their capabilities in organic matter decomposition, soil structure improvement, and fertilization. One case of *Nonomuraea* has been previously reported from bronchoalveolar lavage in an immunosuppressed patient with pneumonia, but the link between *Nonomuraea* and pneumonia was obscured by the presence of other microbes [23]. Recently, another report described a human blood infection with *Nonomuraea*. Though no direct evidence was provided, *N. dietziae* was suspected to be pathogenic in this case, as it was the only microorganism cultured from the patient’s blood [24].

Modern polyphasic taxonomy including whole genome sequence comparisons has led to the reclassification of several complex heterogenous taxa, distinguish between closely related species, correct the classification of misclassified bacteria, and uncovering cryptic diversity [25,26]. Recently, numerous bacterial species have been reclassified based on whole-genome sequence analyses, reflecting advances in genomic-based taxonomy [27–29]. Advanced prokaryotic systematics is crucial for understanding evolution of bacteria with biotechnological, clinical, ecological and agricultural interests, as misclassification can affect disease diagnosis, antibiotic resistance studies, and bioprospecting efforts. Moreover, refining bacterial taxonomy facilitates more precise communication among researchers and enhances our knowledge on microbial evolution and function in diverse ecosystems. Despite these advances, recent species descriptions in the genus *Nonomuraea* have often lacked whole-genome sequencing (WGS) or, when included, have failed to incorporate all species with validly published names in the analyses. Consequently, this has led to erroneous taxonomic conclusions, necessitating the reclassification of certain species.

To clarify these taxonomic uncertainties, we employed digital DNA-DNA hybridization (dDDH), average nucleotide identity (FastANI), average amino acid identity (AAI), percentage of conserved proteins (POCP) analyses, and genome-based phylogeny to reassess the taxonomic status of the genus *Nonomuraea*. These values were evaluated against established thresholds for bacterial species and subspecies differentiation. Whole-genome sequencing analysis provides a resolution to the previously unclear taxonomic status of six *Nonomuraea* species with validly published names.

## Materials and methods

### Genomic dataset

We performed taxonomic analyses based on genomic data including digital DNA-DNA hybridization (dDDH), average nucleotide identity (FastANI), average amino acid identity (AAI), and percentage of conserved proteins (POCP). Genome sequences for all analyzed *Nonomuraea* species and subspecies with validly published names were obtained from the National Center for Biotechnology Information (NCBI) GenBank database, and a summary of their genomic features is presented in S1 Table. Genome completeness and potential contamination were evaluated using CheckM (v1.2.2) [30]. This article does not contain any studies with human participants and/or animals performed by any of the authors. The formal consent is not required in this study.

### Phylogenetic relationship reconstructions

Whole genome-based phylogenetic trees were constructed using the Type (Strain) Genome Server (TYGS), an open-access bioinformatics platform available at https://tygs.dsmz.de [31,32]. To infer phylogenomic relationships, pairwise comparisons of all genomes were performed using the Genome BLAST Distance Phylogeny (GBDP) approach. Intergenomic distances were estimated with the ‘trimming’ algorithm and distance formula *d*_*5*_ [33], with 100 replicates for distance calculations. These distances were then utilized to generate a balanced minimum evolution tree using FASTME 2.1.6.1. Branch support values were derived from 100 pseudo-bootstrap replicates [34].

In addition, core genes-based phylogenomic tree was also constructed. The Roary 3.13.0 pangenome pipeline was utilized to analyze the genomes of selected type strains and identify the different genes, including core, accessory, and strain-unique genes [35]. PRANK v.140110 [36], with a 95% identity cut-off, was employed to generate a core gene alignment and a matrix indicating the presence or lack of genes through Roary. From the core gene alignments, a phylogenomic tree was designed based on the maximum-likelihood method in MEGA11 [37], with the tree replicated 1000 replicates to assess tree robustness. The trees were rooted using *Saccharothrix algeriensis* DSM 44581^T^ as the outgroup. The National Center for Biotechnology Information (NCBI) accession numbers of the sequences used for these analyses are shown (S1 Table).

The 16S rRNA gene sequences were retrieved from the EzBioCloud database (https://www.ezbiocloud.net) using the accession numbers provided in the original publications or from the repositories where the type strains were deposited (S1 and S2 Figs). Evolutionary analyses were conducted in MEGA11 [37]. Multiple sequence alignments were generated using CLUSTAL W (default parameters) [38]. Tree robustness was assessed by bootstrap analysis with 1000 replications [39], using the maximum-likelihood method based on the Tamura-Nei model (S1 and S2 Figs).

### Sequences similarity scores

All pairwise comparisons among the set of genomes were conducted using GBDP and accurate intergenomic distances inferred under the algorithm ‘trimming’ and distance formula *d*_*5*_ [33], 100 distance replicates were calculated each. Digital DNA-DNA hybridization (dDDH) values and confidence intervals were calculated using the recommended settings of the Genome-to-Genome Distance Calculation (GGDC) 4.0 utilizing the recommended BLAST algorithm [33]. Non-symmetrical average nucleotide identity (ANI) scores were calculated using FastANI version 1.3 [40]. To delimit species and subspecies using dDDH values, the thresholds of 70 and 79%, were considered, respectively [41]. To delimit species using FastANI value, the threshold of 95–96% was considered [40]. Furthermore, average amino acid identity (AAI) and percentage of conserved proteins (POCP) were also calculated. AAI values were determined using the online calculator provided by Rodriguez-R and Konstantinidis [42]; and POCP values were calculated using the publicly available code from github.com/hoelzer/pocp, following the ortholog identification thresholds established by Qin et al. [43]. We applied the 50% POCP threshold to our dataset [43]; and a 95–96% AAI threshold [44].

## Results and discussion

### 16S rRNA gene based phylogenetic analyses and sequence comparisons

Pairwise sequence comparisons of almost complete 16S rRNA gene sequences of studied *Nonomuraea* strains (S2 Table), revealed a high degree of genetic similarity across *N. roseoviolacea* subsp. *carminata* DSM 44170ᵀ and *N. roseoviolacea* subsp. *roseoviolacea* JCM 3145ᵀ (99.2%); *N. recticatena* JCM 6835ᵀ, *N. roseola* JCM 3323ᵀ, and *N. dietziae* DSM 44320ᵀ (99.3–99.4%); as well as *N. ferruginea* DSM 43553ᵀ and *N. harbinensis* CGMCC 4.7106ᵀ (99.1%) (S1 Fig). The 16S rRNA gene similarity values are coherent with the close phylogenetic relationship of the type strains listed above, which formed two well-supported subclades in the maximum-likelihood tree (S2 Fig). However, it has been shown in several studies that this gene marker provides little resolution to effectively differentiate closely related taxa [45,46], as reflected in the strong genetic relatedness among the type strains of certain *Nonomuraea* species and subspecies, including *N. roseoviolacea* subsp. *carminata* DSM 44170ᵀ and *N. roseoviolacea* subsp. *roseoviolacea* JCM 3145ᵀ (99.2%); *N. recticatena* JCM 6835ᵀ, *N. roseola* JCM 3323ᵀ, and N*. dietziae* DSM 44320ᵀ (99.3–99.4%); as well as *N. ferruginea* DSM 43553ᵀ and *N. harbinensis* CGMCC 4.7106ᵀ (99.1%). These findings prompted a re-evaluation of the genus taxonomy using whole-genome sequencing, which enables more precise phylogenetic resolution and improved taxonomic classification.

### Genome based phylogenomic analyses and sequence comparisons

**Fig 1:**
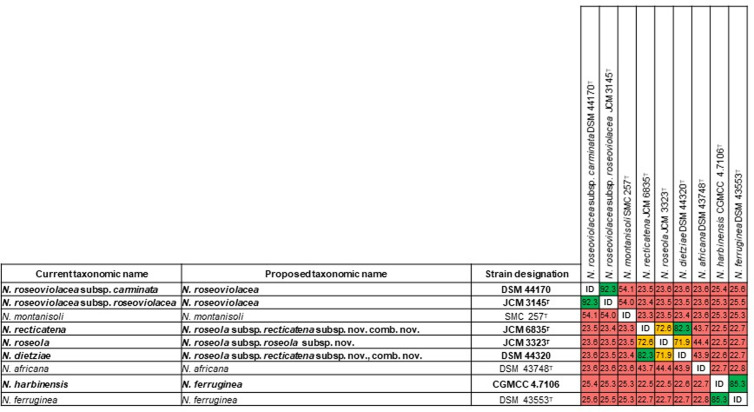
Pairwise comparison of digital DNA-DNA hybridization (dDDH) scores (%) among selected *Nonomuraea* species and subspecies. Accession numbers of gene sequences are provided in S1 Table. Color highlights indicate species and subspecies delineation: dDDH values below 70% (red) suggest different species, values between 70% and 79% (orange) suggest different subspecies within the same species, and values above 79% (green) suggest the same subspecies.

**Fig 2:**
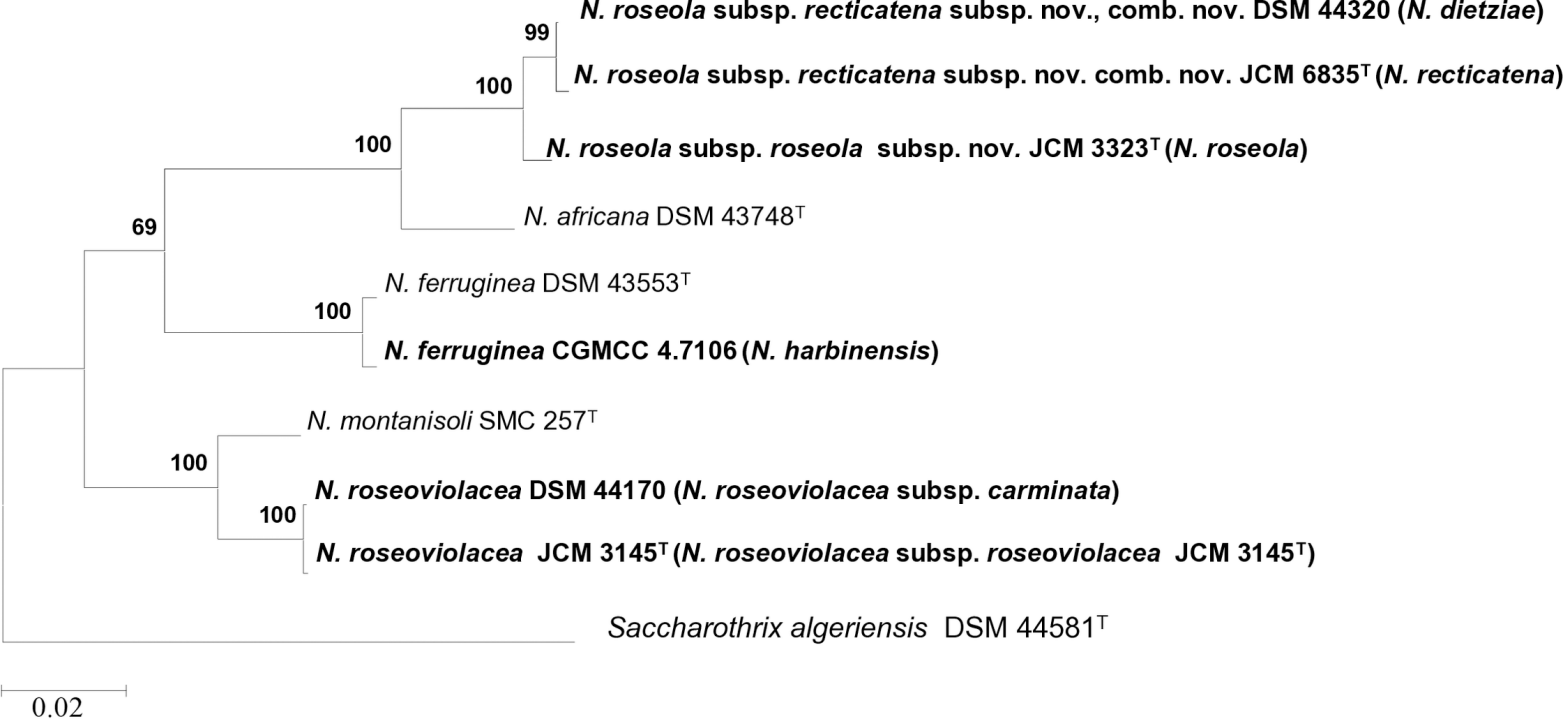
Maximum-likelihood core genes phylogenomic tree of proposed taxonomic names of *Nonomuraea* species and subspecies constructed using the Roary pangenome pipeline [35] and MEGA software version 11 [37] with 1000 bootstrap replications to assess statistical support. This illustrates the evolutionary relationship between the species of *Nonomuraea*. Scientific names shown in parenthesis correspond to the current scientific names. Names in bold are to indicate the proposed taxonomic changes. *Saccharothrix algeriensis* DSM 44581^T^ was used as an outgroup. The scale bar represents 0.02 nucleotide substitutions per sequence position (site). Accession numbers of the genome sequences used for the reconstruction are shown in S1 Table.

To refine the taxonomy of certain *Nonomuraea* species and subspecies, we reconstructed their phylogenetic relationships using whole-genome sequences, focusing on taxa with potential reclassification (S1 Table). We observed a clear phylogenetic separation between some of the species validly named (Figs 1 and S3). However, according to the tree topology and the sequences similarity scores, the taxonomic status of some of them should be revised (Figs 1 and 2, S3 and S4 Figs).

The dDDH (92.3%) and FastANI (99.2%) values between *N. roseoviolacea* subsp. *carminata* DSM 44170^T^ and *N. roseoviolacea* subsp. *roseoviolacea* JCM 3145^T^ are above the defined threshold of 70% and 95–96% for bacterial species demarcation, respectively [47–50]. These findings confirm the assignment of these subspecies to the same species, *N. roseoviolacea* (= ATCC 27297^T^ = BCRC 13406^T^ = CBS 260.72^T^ = CCM 3491^T^ = CCRC 13406^T^ = CGMCC 4.1072^T^ = CIP 106924^T^ = DSM 43144^T^ = IFO 14098^T^ = IMET 9751^T^ = JCM 3145^T^ = KCTC 9283^T^ = NBRC 14098^T^ = NCIB 11117^T^ = NCIMB 11117^T^ = NRRL B-16127^T^ = VKM Ac-909^T^).

The dDDH and FastANI values between *N. recticatena* JCM 6835^T^ and *N. roseola* JCM 3323^T^ are 72.6% and 96.5%, while *N. roseola* JCM 3323^T^ and *N. dietziae* DSM 44320^T^ share 71.9% and 96.6% similarity. However, *N. dietziae* DSM 44320^T^ and *N. recticatena* JCM 6835^T^ exhibit dDDH and FastANI values of 82.3% and 97.7%, respectively. These values are well above the designated threshold listed above for bacterial species delineation, supporting the classification of the three strains as the same species. Furthermore, the dDDH value of 82.3% between *N. dietziae* DSM 44320^T^ and *N. recticatena* JCM 6835^T^ surpasses the 79% cutoff proposed by Meier-Kolthoff et al. [41] for subspecies delineation, indicating that these two strains belong to the same subspecies.

Therefore, we propose the establishment of two subspecies: *N. roseola* subsp. *roseola* subsp. nov., with ATCC 33579^T^ = DSM 43767^T^ = DSM 44323^T^ = IFO 14685^T^ = IMET 9576^T^ = INA 1671^T^ = JCM 3323^T^ = KCTC 9282^T^ = NBRC 14685^T^ = VKM Ac-1180^T^ as the type strain, and *N. roseola* subsp. *recticatena* subsp. nov., comb. nov., with DSM 43937^T^ = IFO 14525^T^ = INA 308^T^ = JCM 6835^T^ = KCTC 9279^T^ = NBRC 14525^T^ = VKM Ac-940^T^ as the type strain (Fig 1). Thus, the type strain of *N. dietziae* (Stackebrandt et al. 2001) become a non-type strain of *N. roseola* subsp. *recticatena* subsp. nov., comb. nov.

The dDDH and FastANI values between *N. ferruginea* DSM 43553^T^ and *N. harbinensis* CGMCC 4.7106^T^ are 85.3% and 98.3%, respectively, suggesting they belong to the same species. As *N. ferruginea* (Meyer 1981) Zhang et al. 1998 has nomenclatural priority over *N. harbinensis* Wang et al. 2017, *N. harbinensis* species should be regarded as a later heterotypic synonym of *N. ferruginea* (Fig 1). All the proposed taxonomic changes (S1 Table) are supported by additional Overall Genome Relatedness Indices (OGRIs), including FastANI (S4 Fig), AAI (S5 Fig), and POCP values (S6 Fig).

### Taxonomic conclusions

Considering the genetic divergence observed, we propose the following taxonomic revisions: (i) *N. roseoviolacea* subsp. *carminata* and *N. roseoviolacea* subsp. *roseoviolacea* should be elevated to species rank *as N. roseoviolacea*; (ii) *N. recticatena*, *N. dietziae* and *N. roseola* should be reclassified as subspecies within *N. roseola*, leading to the establishment of *N. roseola* subsp. *roseola* subsp. nov., and *N. roseola* subsp. *recticatena* subsp. nov. comb. nov. (encompassing both *N. recticatena* and *N. dietziae*), and (iii) *N. harbinensis* should be designated as later heterotypic synonyms of *N. ferruginea*.

### Future directions

The discovery of novel *Nonomuraea* strains holds significant potential for biotechnological, medical, agricultural, and environmental applications due to their diverse metabolic capabilities. These species contribute to soil health by facilitating organic matter decomposition and nutrient cycling, thereby supporting agricultural productivity [51]. Certain *Nonomuraea* strains also enhance plant growth and provide resistance against pathogens, making them valuable in sustainable agriculture [5]. Several *Nonomuraea* species have demonstrated biotechnological potential. *N. aurantiaca* has been identified as a cellulase producer, aiding biomass degradation [3], while *N. aridisoli* shows promise for synthesizing secondary metabolites with pharmaceutical applications [2]. The genus is also a source of structurally unique antibiotics with strong bioactivities, particularly against resistant bacterial strains. *N. jiangxiensis* produces nine thiopeptides with potent antimicrobial activity against Gram-positive bacteria, including *Staphylococcus aureus*, with MIC90 values ranging from 2 µM to 11 µM [18]. Additionally, *Nonomuraea gerenzanensis* synthesizes A40926, a glycopeptide antibiotic precursor to dalbavancin [52], with genetic modifications improving its production by over 30% [53]. *Nonomuraea coxensis* has been found to produce a novel glycopeptide antibiotic related to A40926, further highlighting the diversity of glycopeptides within the genus [54]. Moreover, a rare *Nonomuraea* species has been reported to synthesize five unique *β*-carboline derivatives, termed nonocarbolines A–E. Among them, nonocarboline B exhibits moderate antifungal activity against the microfungus *Mucor hiemalis*, while nonocarboline D demonstrates significant cytotoxicity against A-549 human lung carcinoma cells [55]. Beyond glycopeptides, *Nonomuraea* species produce various antibiotics, including macrolides, cyclopeptides, and thiazole derivatives. Karamomycins A–C, isolated from *Nonomuraea endophytica*, have shown cytotoxic properties, further expanding the range of bioactive compounds derived from this genus [56].

#### Protologues.

**Description of *Nonomuraea roseola* subsp. *roseola* subsp. nov.:**

(ro.se’o.la. L. masc. adj. *roseus*, rose-colored; L. fem. suff. *-ola*, diminutive ending; N.L. fem. adj. *roseola*, intended to mean with a rosy tinge, referring to the rose-colored aerial mycelium). Following our proposition to reclassify *Nonomuraea roseola* as *Nonomuraea roseola* subsp. *roseola* subsp. nov., the description of this subspecies is identical to the description given by Zhang et al. [1], with the following additions. The G + C content of the type-strain genome is 70% and its approximate size is 10.4 Mbp. The GenBank accession number is GCA_039535395.1. The type strain is JCM 3323^T^ = ATCC 33579^T^ = DSM 43767^T^ = DSM 44323^T^ = IFO 14685^T^ = IMET 9576^T^ = INA 1671^T^ = KCTC 9282^T^ = NBRC 14685^T^ = VKM Ac-1180^T^.

**Description of *Nonomuraea roseola* subsp. *recticatena* subsp. nov. comb. nov.:**

(rec.ti.ca.te.na. L. masc. perf. part. *rectus*, straight; L. fem. n. *catena*, a chain; N.L. fem. n. *recticatena*, a straight chain (nominative in apposition). Following our proposition to create *Nonomuraea roseola* subsp. *roseola* subsp. nov., and to reclassify *Nonomuraea recticatena* as *Nonomuraea roseola* subsp. *recticatena* subsp. nov., comb. nov., and the description of this subspecies follows Zhang et al. [1], with the following additions. The G + C content of the type-strain genome is 70% and its approximate size is 10.5 Mbp. The GenBank accession number is GCA_039533245.1. The type strain is JCM 6835^T^ = DSM 43937^T^ = IFO 14525^T^ = INA 308^T^ = KCTC 9279^T^ = NBRC 14525^T^ = VKM Ac-940^T^.

**Emended description of *Nonomuraea ferruginea* corrig. (Meyer 1981) Zhang et al. 1998:**

(fer.ru.gi’ne.a. L. fem. adj. *ferruginea*, of an iron gray color, rusty brown, referring to the orange-brown-colored substrate mycelium). Following our proposition to reclassify *Nonomuraea harbinensis* as a later heterotypic synonym of *Nonomuraea ferruginea*, the description of this species is identical to the description given by Zhang et al. [1], with the following additions. The G + C content of the type-strain genome is 71.5% and its approximate size is 9.2 Mbp. The GenBank accession number is GCA_027620355.1. The type strain is 14094^T^ = ATCC 35575^T^ = BCRC 12537^T^ = CCM 3424^T^ = CCRC 12537^T^ = CIP 106925^T^ = DSM 43553^T^ = IFO 14094^T^ = IMET 9567^T^ = JCM 3283^T^ = KCTC 9269^T^ = NBRC 14094^T^ = NCIB 11630^T^ = NCIMB 11630^T^ = NRRL B-16096^T^ = VKM Ac-854^T^.

**Emended description of *Nonomuraea roseoviolacea* corrig. (Nonomura and Ohara 1971) Zhang et al. 1998:** (ro.se.o.vi.o.la.ce.a. L. masc. adj. *roseus*, rosy; L. adj. *violaceus -a -um*, violet colored; N.L. fem. adj. *roseoviolacea*, rosy, violet colored, referring to the color of the substrate mycelium). As we propose to elevate *Nonomuraea roseoviolacea* subsp. *roseoviolacea* (Nonomura and Ohara 1971) Gyobu and Miyadoh 2001 and *Nonomuraea roseoviolacea* subsp. *carminata* (Gauze et al. 1973) Gyobu and Miyadoh 2001 to the species level, the description of *N. roseoviolacea* follows Zhang et al. [1], with the following additions: G + C content of the type-strain genome is 72.5% and its approximate size is 9.8 Mbp. The GenBank accession number is GCA_039532505.1. The type strain is JCM 3145^T^ = ATCC 27297^T^ = BCRC 13406^T^ = CBS 260.72^T^ = CCM 3491^T^ = CCRC 13406^T^ = CGMCC 4.1072^T^ = CIP 106924^T^ = DSM 43144^T^ = IFO 14098^T^ = IMET 9751^T^ = KCTC 9283^T^ = NBRC 14098^T^ = NCIB 11117^T^ = NCIMB 11117^T^ = NRRL B-16127^T^ = VKM Ac-909^T^.

## Supporting information

S1 Fig Pairwise comparison of 16S rRNA scores (%) among selected *Nonomuraea* species and subspecies. Accession numbers of gene sequences used are shown in the phylogenetic tree of S2 Fig and S2 Table. Color highlights indicate species delineation: 16S rRNA similarity below 98.65% (red) suggests different species, while values above 98.65% (orange) suggest probable conspecificity.
(TIF)

S2 Fig Maximum-likelihood phylogenetic tree reconstructed from almost complete 16S rRNA gene sequences of all *Nonomuraea* child taxa. The evolutionary history was inferred by using the maximum-likelihood method based on the Tamura-Nei model. Numbers at the nodes are bootstrap values, expressed as a percentage of 1000 resamplings (only values > 50% are shown). Evolutionary analyses were conducted in MEGA11 based on 100 replications. Accession numbers of used gene sequences are shown in parenthesis. *Saccharothrix algeriensis* DSM 44581^T^ was used as an outgroup. Bar 0.01 nucleotide substitution per site.
(TIF)

S3 Fig Phylogenomic tree based on whole-genome sequences of *Nonomuraea* in the TYGS tree inferred with FastME 2.1.6.1 [31,34], from the Genome BLAST Distance Phylogeny approach (GBDP); distances were calculated from genome sequences. The branch lengths are scaled in terms of GBDP distance formula *d*_*5*_. The numbers above the branches are GBDP pseudo-bootstrap support values > 70% from 100 replications. The tree was rooted at the midpoint, and *Saccharothrix algeriensis* DSM 44581^T^ was used as an outgroup. The same colour indicates the same species Cluster. NCBI accession numbers of the sequences used for the analyses are shown in S1 Table.
(TIF)

S4 Fig Pairwise comparison of digital Average Nucleotide Identity (FastANI) scores (%) among selected *Nonomuraea* species and subspecies. Accession numbers of gene sequences used are provided in S1 Table. Color highlights indicate species: FastANI values below 95% (red) indicate different species, and values above 96% (green) indicate the same species.
(TIF)

S5 Fig Pairwise comparison of Average Amino Acid Identity (AAI) scores (%) among selected *Nonomuraea* species and subspecies. Accession numbers of genome sequences are provided in S1 Table. Color highlights indicate species: AAI values below 95% (red) indicate different species, and values above 96% (green) indicate the same species.
(TIF)

S6 Fig Pairwise comparison of POCP (Percentage of Conserved Proteins) scores (%) among selected *Nonomuraea* species and subspecies. Accession numbers of genome sequences are provided in S1 Table. Color highlights species and subspecies delineation: POCP values below 50% (red) suggest strains belong to different genera, and values above 50% (green) suggest strains belong to the same genus.
(TIF)

S1 Table Features of the genome sequences analyzed in this study. (XLS)

S2 Table Characteristics of the 16S rRNA gene sequences used in this study. (DOCX)
