# Supplementary figures and images for: Whole genome-based reclassification of several species of the genus Nonomuraea
Source: PLoS One. 2025 Jul 1;20(7):e0327003. doi: 10.1371/journal.pone.0327003 (PMC12212573; doi:10.1371/journal.pone.0327003)

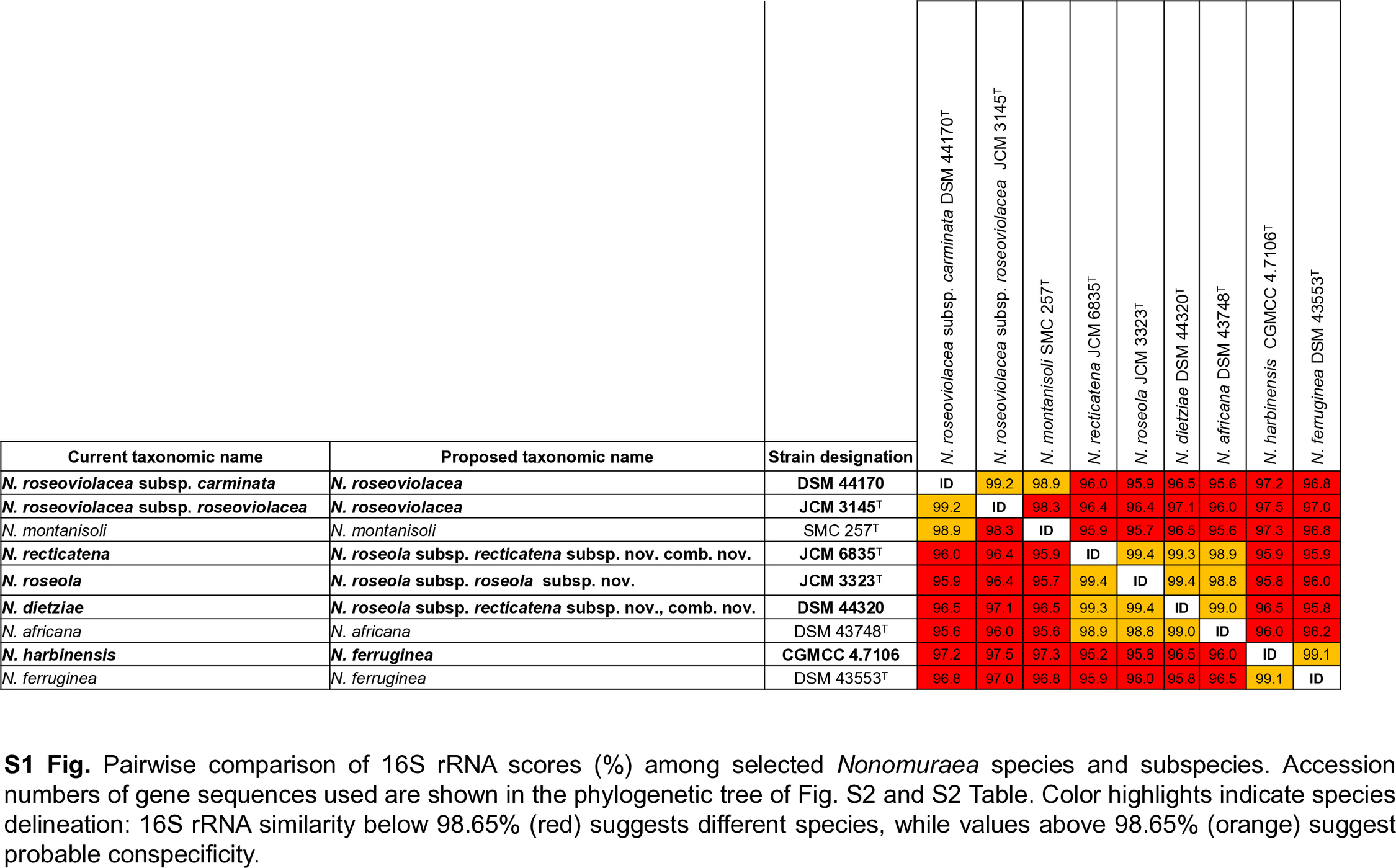

Supplement: S1 Fig — Accession numbers of gene sequences used are shown in the phylogenetic tree of S2 Fig and S2 Table. Color highlights indicate species delineation: 16S rRNA similarity below 98.65% (red) suggests different species, while values above 98.65% (orange) suggest probable conspecificity. (TIF) [file pone.0327003.s001.tif]

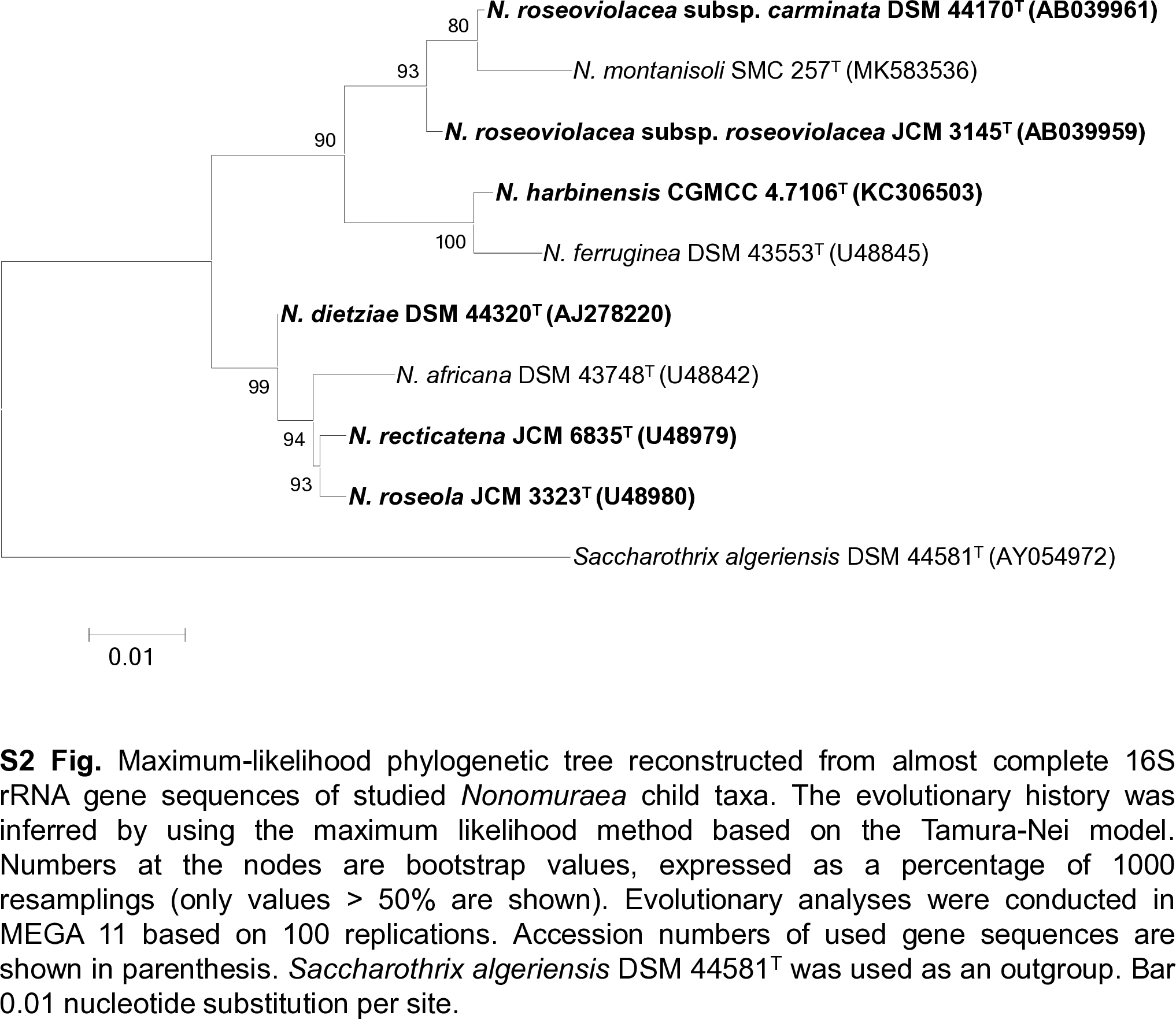

Supplement: S2 Fig — The evolutionary history was inferred by using the maximum-likelihood method based on the Tamura-Nei model. Numbers at the nodes are bootstrap values, expressed as a percentage of 1000 resamplings (only values > 50% are shown). Evolutionary analyses were conducted in MEGA11 based on 100 replications. Accession numbers of used gene sequences are shown in parenthesis. Saccharothrix algeriensis DSM 44581T was used as an outgroup. Bar 0.01 nucleotide substitution per site. (TIF) [file pone.0327003.s002.tif]

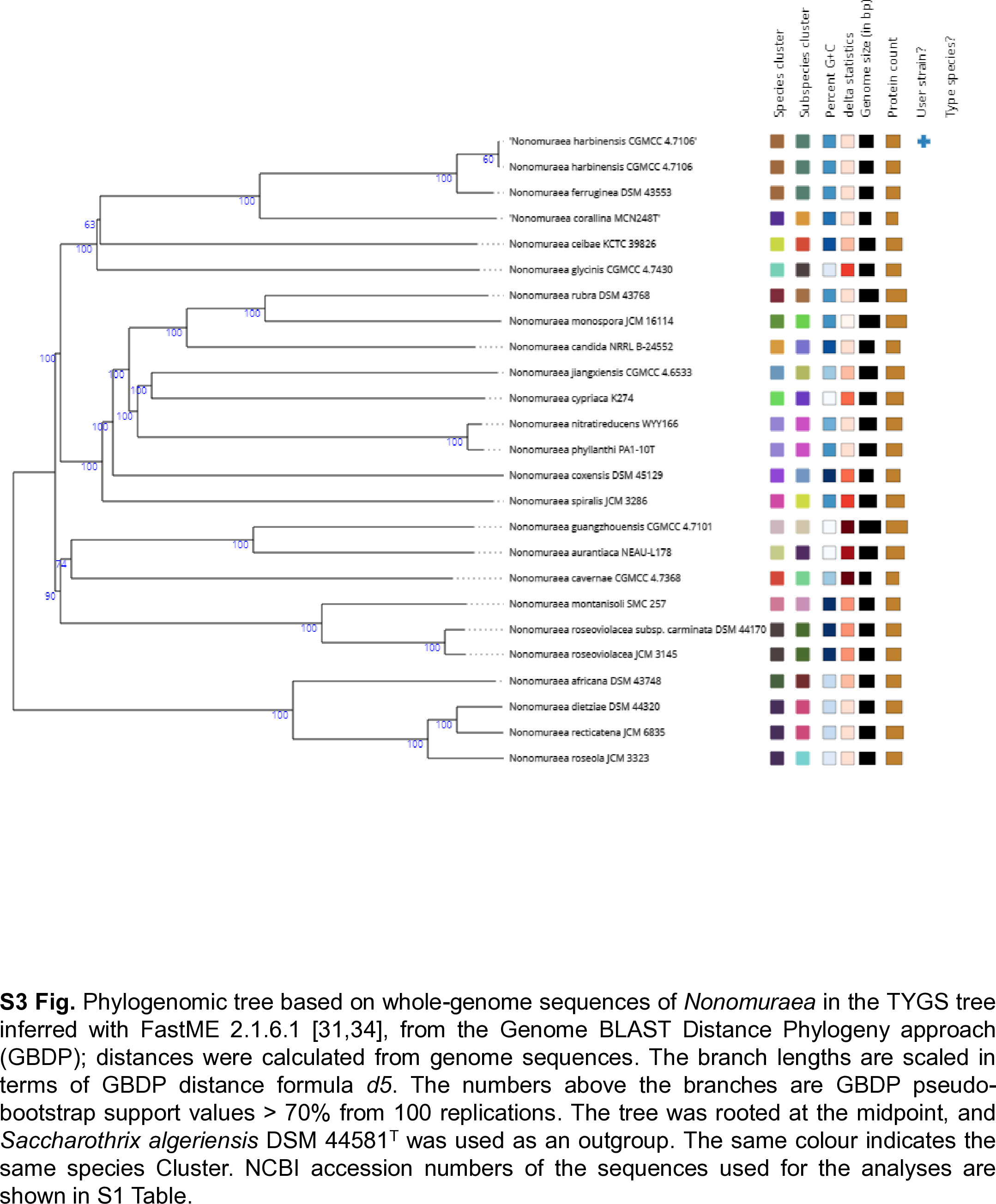

Supplement: S3 Fig — The branch lengths are scaled in terms of GBDP distance formula d5. The numbers above the branches are GBDP pseudo-bootstrap support values > 70% from 100 replications. The tree was rooted at the midpoint, and Saccharothrix algeriensis DSM 44581T was used as an outgroup. The same colour indicates the same species Cluster. NCBI accession numbers of the sequences used for the analyses are shown in S1 Table. (TIF) [file pone.0327003.s003.tif]

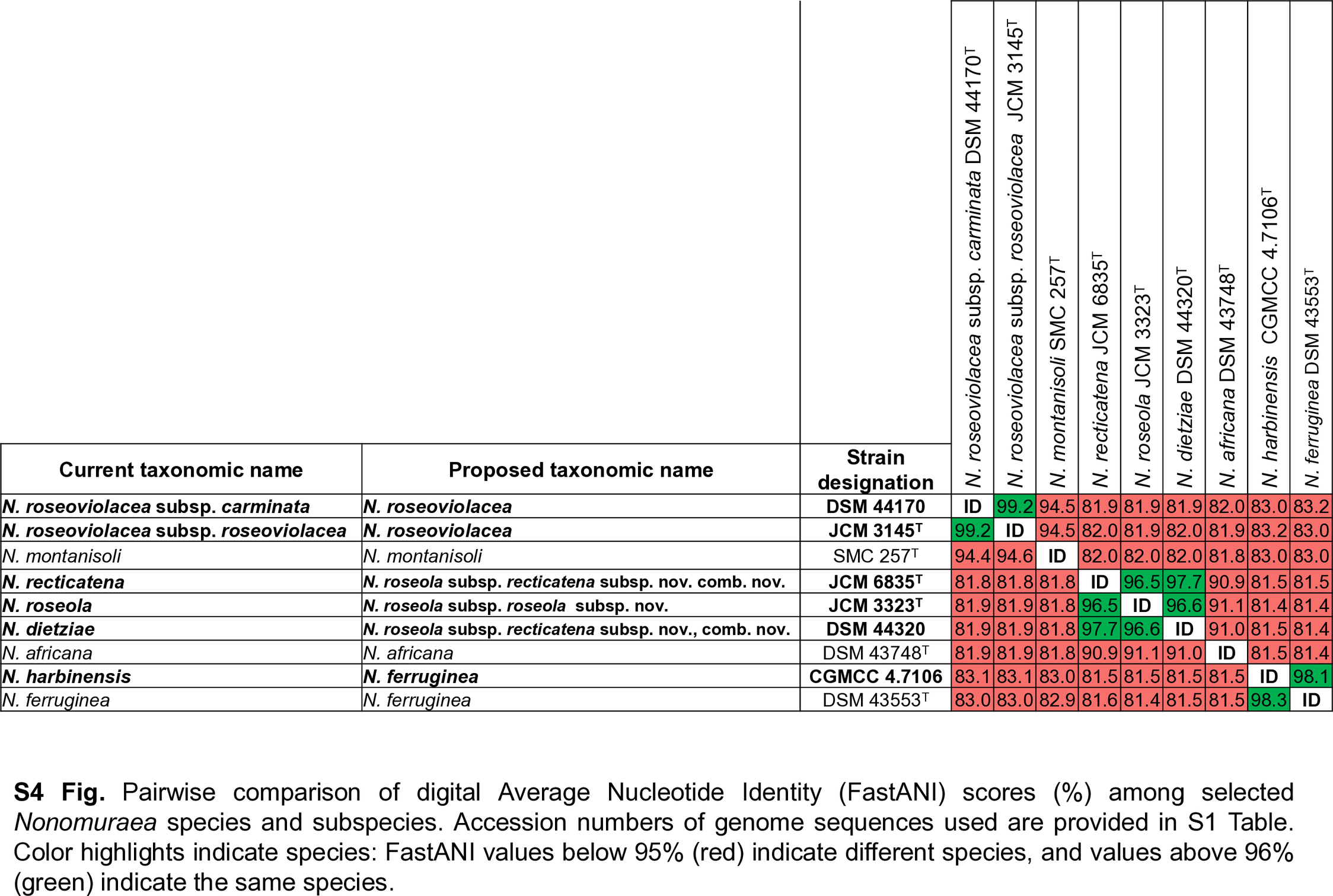

Supplement: S4 Fig — Accession numbers of gene sequences used are provided in S1 Table. Color highlights indicate species: FastANI values below 95% (red) indicate different species, and values above 96% (green) indicate the same species. (TIF) [file pone.0327003.s004.tif]

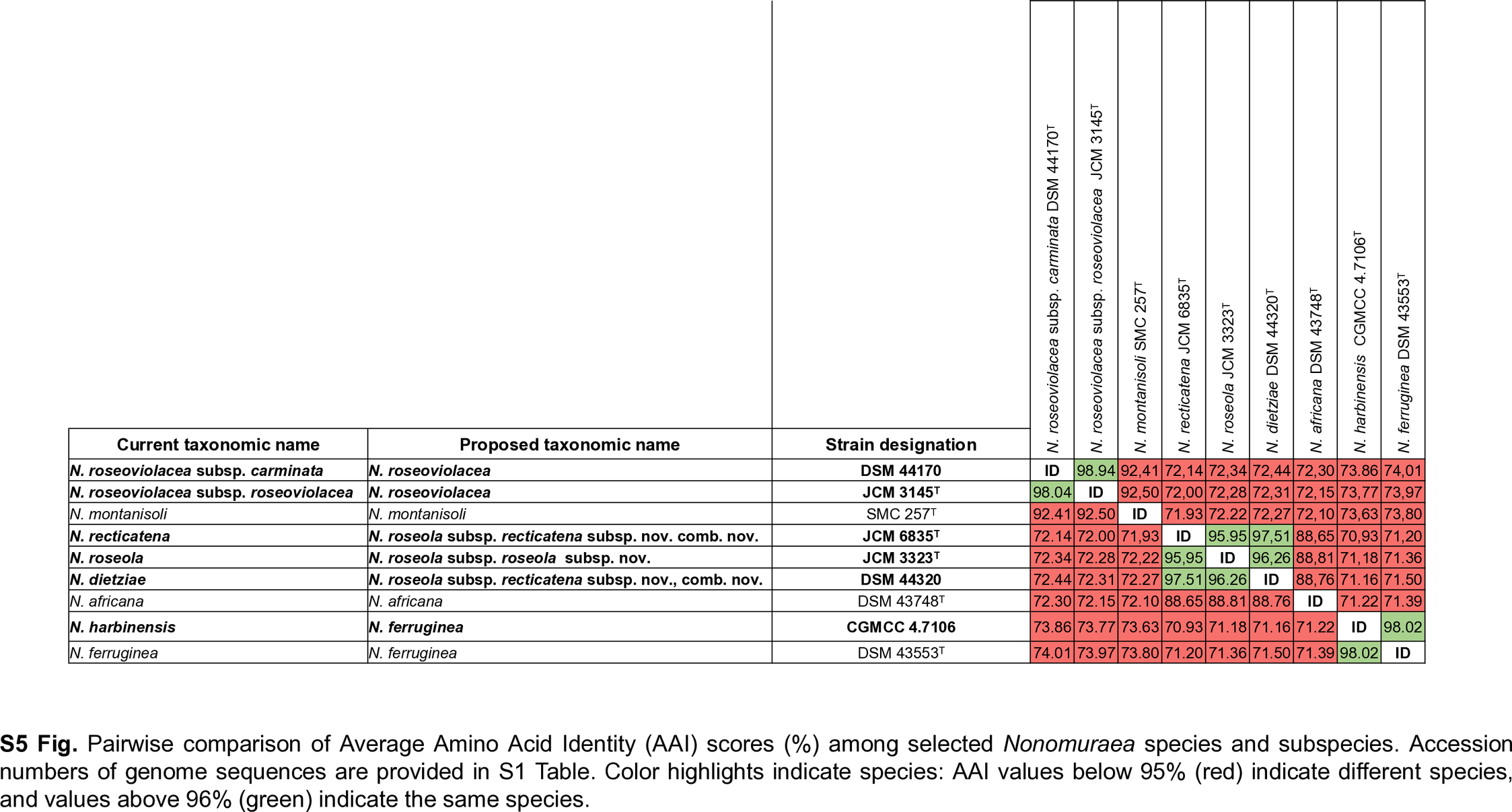

Supplement: S5 Fig — Accession numbers of genome sequences are provided in S1 Table. Color highlights indicate species: AAI values below 95% (red) indicate different species, and values above 96% (green) indicate the same species. (TIF) [file pone.0327003.s005.tif]

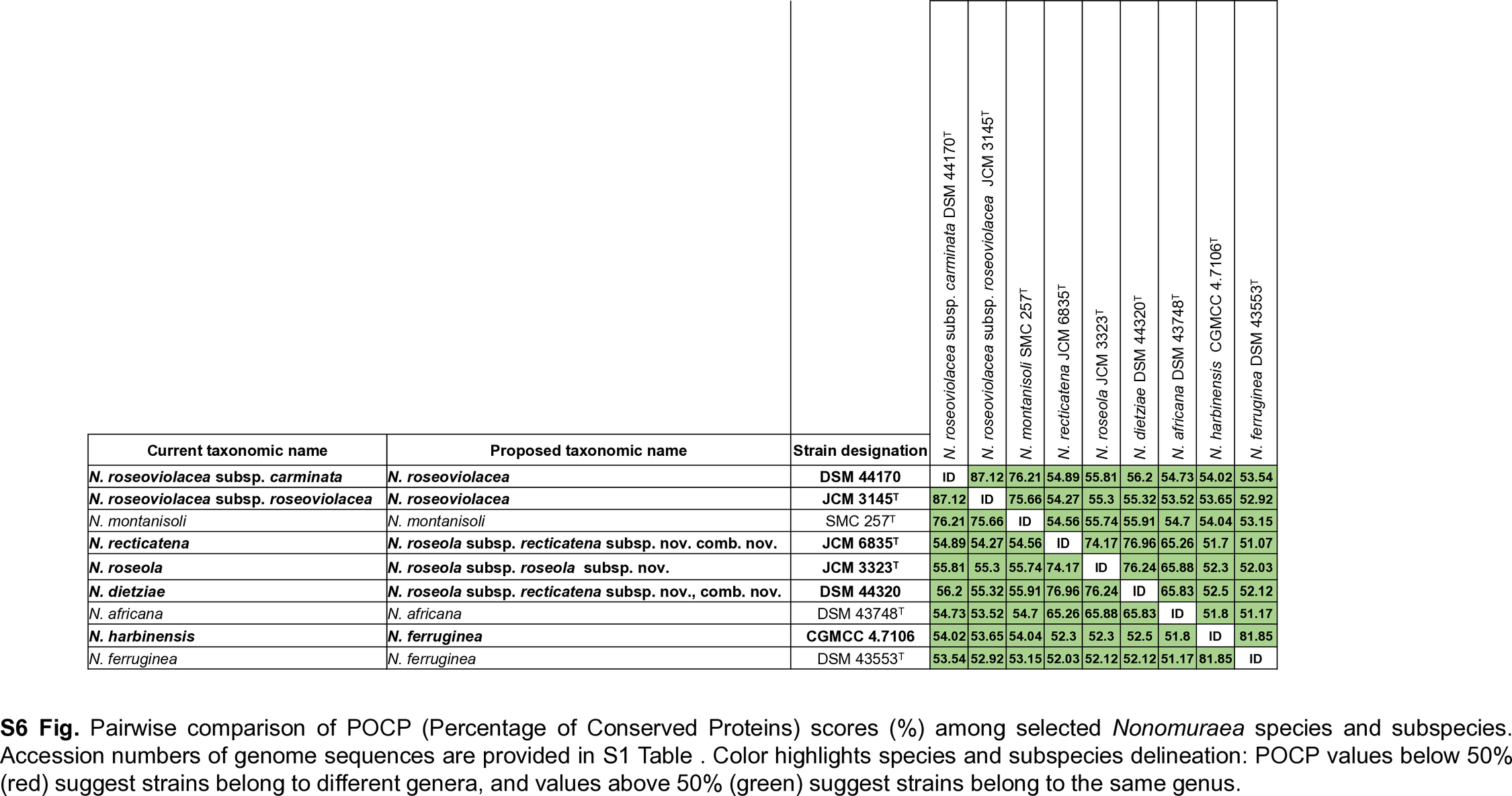

Supplement: S6 Fig — Accession numbers of genome sequences are provided in S1 Table. Color highlights species and subspecies delineation: POCP values below 50% (red) suggest strains belong to different genera, and values above 50% (green) suggest strains belong to the same genus. (TIF) [file pone.0327003.s006.tif]
