# Supplementary material for: Whole genome-based reclassification of several species of the genus Nonomuraea
Source: PLoS One. 2025 Jul 1;20(7):e0327003. doi: 10.1371/journal.pone.0327003 (PMC12212573; doi:10.1371/journal.pone.0327003)
Supplement: S2 Table — (DOCX) [file pone.0327003.s008.docx]

**S2** **Table** Characteristics of the 16S rRNA gene sequences used in this study.

| **Current taxonomic name** | **Proposed taxonomic name** | **Strain designations** | **Accession number** | **Length (pb)** | **16S rRNA aligned sequence length (bp) after trimming** |
| --- | --- | --- | --- | --- | --- |
| ***N. roseoviolacea* subsp. *carminata*** | ***N. roseoviolacea*** | IFO 15903 | AB039961 | 1384 | 1384 |
| ***N. roseoviolacea* subsp. *roseoviolacea*** | ***N. roseoviolacea*** | IFO 14098^T^ | AB039959 | 1437 | 1383 |
| *N. montanisoli* | *N. montanisoli* | SMC 257^T^ | MK583536 | 1444 | 1384 |
| ***N. recticatena*** | **N. *roseola* subsp. *recticatena* subsp. nov. comb. nov.** | IFO 14525^T^ | U48979 | 1413 | 1381 |
| ***N. roseola*** | **N. *roseola* subsp. *roseola* subsp. nov.** | IFO 14685^T^ | U48980 | 1413 | 1381 |
| ***N. dietziae*** | **N. *roseola* subsp. *recticatena* subsp. nov., comb. nov.** | DSM 44320 | AJ278220 | 1438 | 1384 |
| *N. africana* | *N. africana* | IFO 14745 | U48842 | 1413 | 1381 |
| ***N. harbinensis*** | ***N. ferruginea*** | NEAU-yn31 | KC306503 | 1447 | 1387 |
| *N. ferruginea* | *N. ferruginea* | IFO 14094^T^ | U48845 | 1415 | 1383 |
| *Saccharothrix algeriensis* | *Saccharothrix algeriensis* | NRRL B-24137^T^ | AY054972 | 1429 | 1429 |
